# India’s Opportunity to Address Human Resource Challenges in Healthcare

**DOI:** 10.7759/cureus.40274

**Published:** 2023-06-11

**Authors:** Sangeeta G Saxena, Thomas Godfrey

**Affiliations:** 1 Public Health Sciences, Coastal Carolina University, Conway, USA; 2 Public Health Sciences, Penn State College of Medicine, Hershey, USA

**Keywords:** working conditions, indian public health standards, auxiliary nurse midwives, human resource, health sector reform, india, health policy

## Abstract

India’s health indicators have improved in recent times but continue to lag behind those of its peer nations. The country with a population of 1.3 billion, has an estimated active health workers density of doctors and nurses/midwives of 5.0 and 6.0 respectively, for 10,000 persons, which is much lower than the WHO threshold of 44.5 doctors, nurses, and midwives per 10,000 population. The issue is compounded by the skewed inter-state, urban-rural, and public-private sector divide. Calls to urgently augment the skilled health workforce reinforce the central role human resources have in healthcare, which has evolved into a complex multifactorial issue. The paucity of skilled personnel must be addressed if India is to accelerate its progress toward achieving universal health coverage and its sustainable development goals (SDGs).

The recent increase in the federal health budget offers an unprecedented opportunity to do this. This article utilizes the ready materials, extract and analyze data, distill findings (READ) approach to adding to the authors' experiential learning to analyze the health system in India. The growing divide between the public and the burgeoning private health sector systems, with the latter's booming medical tourism industry and medical schools, are analyzed along with the newly minted National Medical Council, to recommend policies that would help India achieve its SDGs.

## Introduction and background

India’s health indicators have improved in recent times but continue to lag behind those of its peer nations. The country has an estimated active health workers’ density much lower than the WHO recommended thresholds [1]. The issue is compounded by the skewed inter-state, urban-rural, and public-private sector divide. The paucity of skilled personnel is a multi-factorial issue and needs to be addressed if India is to accelerate its progress toward achieving universal health coverage and its sustainable development goals (SDGs). 

The authors describe these issues by providing an overview of the public and private sectors and the growing divide between them due to their divergent strategies, with the latter now having a booming medical tourism industry and a burgeoning number of medical schools. They identify the opportunities available within the newly created National Medical Council and the recent increase in the federal health budget [2]. The recommendations made to address the paucity of quality health personnel include the creation of transparent governance, strengthening the health infrastructure, upskilling the existing workforce, and creating partnerships with the much larger private sector. The methodology used is the READ approach [3], which is a systematic approach for document analysis in health policy research, consisting of readying one's materials, extracting the data, and analyzing it to distill the findings. An extensive literature search was performed, and 56 articles published in peer-reviewed journals between 2005 and 2021 were selected and analyzed. The corresponding authors' experiential knowledge served as the foundation for the analysis.

## Review

Overview of the public and private health sectors 

The government-funded health sector, which is the provider of healthcare to vulnerable populations, has been chronically underfunded with 1.28% of the GDP. This translates to a healthcare expenditure of $2.7 per citizen per year. As a consequence, India has 0.7 public hospital beds per 100,000 people [2] now and 0.576 physicians per 1,000 population in 2000 [4], compared to the World Health Organization's recommended doctor-to-population ratio of 1:1,000 [5]. Since the inception of the National Health Mission (NHM) in 2005, the government has aimed to increase the quantum of services provided, but a lack of focus on quality has failed to make a dent in healthcare indicators [6]. At best, 37% of the population had any health insurance coverage in 2018 [7].

This has contributed to the for-profit private health sector becoming the dominant provider of healthcare for it is perceived to provide quality care [8]. It consumes 5.1% of the GDP, which is financed by Out-Of-Pocket (OOP) expenditure. This sector spans a wide range, from world-class health facilities, such as Narayana Health, an internationally accredited, high quality, tertiary healthcare service provider, to individual informal provider clinics, which are establishments providing medical care, often manned by a solo provider who does not have a formal medical qualification or registration. World-class health facilities exist in urban areas and have enabled India to become a leading destination for medical tourism [9]. The informal providers are concentrated in urban slums and rural areas where they are the first choice of care for they have built a long-standing trusted constant presence in their communities and have adapted to their social, economic, and cultural norms. 

Public health sector (NHM) strategies

The NHM has sought to address health challenges through five approaches - communitization, flexible financing, improved management through capacity building, monitoring progress against standards, and innovations in human resource management [10]. Multiple new cadres have been created to provide primary healthcare and accelerate the pace toward universal health coverage. 

A key cadre has been the female community health workers, Accredited Social Health Activists (ASHA), one for every 1,000 population. They number a million [11] to date and serve to increase the reach of the Auxiliary Nurse Midwives (ANM), who were meant to serve a population of 5,000 but in reality, serve up to 20,000 people. This exercise in task shifting from ANMs to ASHAs cadre raises concerns. ANMs are high school graduates who receive 18 months of training while ASHAs attend school up to the eighth grade, sometimes even less, and receive 23 days’ initial training with additional on-the-job need-based short training [12]. ANMs are government employees and receive a salary while the ASHAs are treated as private contractors who receive a payment proportionate to the amount of work performed. A recent synthesis of the evaluation of ASHAs from a health systems perspective shows broader system constraints and few overall positive findings [12].

Other new cadres are being hired by the government on a contractual basis. the Rural Medical Assistants (RMAs) and paramedical personnel. The RMA [13] is a three-year diploma [13,14] course similar to the physician assistant program in the US. The RMAs work at Primary Health Centers and initial evaluation [15] is positive. Several paramedical councils now exist which have a number of courses varying in duration from six to 24 months to train personnel for the provision of supportive care in health facilities and homes. The scale of such initiatives remains nascent and systemic integration pathways remain undefined. 

While ASHAs remain contractors, other cadres, including doctors and nurses, are being hired on a contractual basis. Since the inception of the NHM in 2005, 275,000 contractual personnel have been inducted into the workforce [16]. Doctors and nurses are incentivized to accept contractual posts in rural areas by linking these postings with monetary incentives and preferential admission into residencies. Doctors trained in Indian systems of medicine such as Ayurveda, Siddha, and Unani (AYUSH) are trained in bridge courses in allopathic medicine. Working conditions in the public sector remain demotivating [16], resulting in low retention rates of these personnel and dissatisfaction levels remaining high. This is so because contractual employees have lower remuneration as compared to their regular counterparts, salary payments are delayed, and the temporary nature of contracts remains a cause of concern [17]. The poor infrastructure and supply chain issues [17] demotivate all employees. Further, the increasing incidents of violence by communities against doctors [18,19], especially during the COVID-19 pandemic [20], add insult to injury. 

Such system failings demotivate doctors and encourage them to seek other opportunities. The majority of doctors, (65%) [1], work in the private health sector. This share continues to grow. Others emigrate, with more than 60,000 Indian-born physicians’ now practicing in high-income countries [21]. This means about 10% of the physicians trained in India have left the country, making it the largest émigré physician workforce in the world [21]. 

Another reform brought in in 2018 is “Ayushman Bharat,” a publicly financed health insurance scheme for the poor. This is designed to allow them to access primary care from health and wellness centers and avail secondary and tertiary care services from participating private health facilities [22]. The scheme has the potential [23] to provide stewardship for improved governance and quality control, thereby accelerating India’s progress toward its SDG goals. However, a recent performance evaluation [24] shows the scheme has not succeeded in improving access or providing financial protection. Similar schemes have been launched independently by various states but results, at best have been mixed [25-28]. This raises concerns about the viability of publicly funded purchasing of services from private providers. 

Private health sector strategies

The for-profit private health sector has perceived opportunities in this unmet demand for healthcare services [29]. It has emerged as the preferred provider of care, as patients see it as providing services that are more available and of a higher quality. This trend towards medicalization and corporatization remains largely unregulated [30] with primary care frequently eclipsed by the more expensive secondary and tertiary care [22]. During the eighties and the nineties, the private sector was largely made up of solo practitioners or small nursing homes. Since the turn of the century, the sector has become dominated by corporate houses in urban areas and is rapidly expanding to tier II and III cities. There has been a marked spike in increased OOP expenditure, which accounted for 62% of total health expenditure (US$60.6 billion out of US$ 97.1 billion) in 2014 [31]. This sector attracts patients from around the world, with nearly 234,000 coming into the country in 2015, bringing an influx of $3 billion, making India the third most popular destination worldwide for medical tourism [9].

The private sector has invested heavily in setting up medical colleges which have proven to be profitable. With 619 medical colleges, India has the highest number per population in the world, as of May 21, 2020. Of these, 279 are in the private sector. Their fees are high, as much as 100 times those of government colleges, [32] and consequently, doctors graduating from these colleges are not attracted to the paltry remuneration offered by contractual government posts. They gravitate towards higher remuneration in the private sector, which is consequently the largest employer [1] of doctors in India. In practice, this sector places constraints of a different kind. In an effort to maximize profit, the corporate sector imposes performance targets and practice constraints on doctors’ professional autonomy. A small number of “star” doctors with flourishing practices can set their rules but the majority of young and early career doctors face a simultaneous erosion of their status and opportunity [33].

National Medical Council

India revamped its inept Medical Commission with a National Medical Council (NMC) in 2019 with the aim of improving the regulation of health professionals (doctors and Community Health Providers) and medical education. The NMC Act remains silent about the interplay between the private corporate sector, the pharmaceutical industries, medical education, and healthcare services. Introducing clarity here in tandem with other reforms would ensure there is a coordinated implementation of all the inputs needed for the provision of universal health coverage. The current trend of centralizing control of medical education leaves little leeway for states to adopt medical education to match the geographical, cultural, social, and economic diversity India represents [22].

Recommendations

Sustain the Increase in the Budget for Healthcare

The total expenditure on health in India is 3.84% of its GDP [34]. Compare this to the US with close to 18% devoted to healthcare and one-quarter of the population. The Government of India has allocated about 1.29% of its budget to healthcare in spite of a stated commitment in the National Health Policy 2017 to increase the allocation to 2.5% by 2025. In contrast, the Domestic General Government Health Expenditure was US 8.6%, Brazil 4%, and China's 2.9% [35]. This year the Indian government doubled its allocation to just over $30 billion [36]. With just 1.29% traditionally coming from the government, the rest has come from OOP expenditures by consumers. This has resulted in many families falling into poverty every year paying for healthcare they can ill afford. The Government must increase its share of expenditure to put it further in line with other middle-income countries and its neighbors.

*Improve Efficiency* 

The public health system is currently unable to utilize even the 1% of the GDP that has traditionally been available to it. With a doubling of the federal government’s allocation in 2022, its capacity to do so must be increased. Increasing the financial allocation and its utilization capacity will enhance the health infrastructure and workforce capability. This requires institutional capacity building and competency-based training in the government healthcare system [37]. This will allow the country to align its health services to local priorities as India exhibits its diversity in its population and disease profiles. The recently started National Health Protection Mission has limited uptake The majority of Indians (up to 80.9% in urban and 85.9% in rural) still do not have health insurance [38].

Accredit Health Facilities and in Practice Regulate the Private Health Sector

The public health sector has revised standards [39] for healthcare infrastructure, personnel, equipment and services for primary, secondary and tertiary care. They need to be utilized to accredit health facilities in order to achieve standardization of the health system. The private health sector is the major provider of health care in India [40]. It consists of the entire spectrum of single provider clinics run by unqualified staff to state-of-the-art hospitals. The latter provide world class care and have made India one of the major hubs for medical tourism [41,42]. However, this sector remains largely unregulated. The limited rules that exist are rarely enforced. For example, these state-of-the-art corporate hospitals were allocated land in prime urban locations on the premise that a set percentage of patients from economically weaker off sections of society would receive care at little-to-no-cost. This is not often the case. The government could take steps to ensure the existing rules are implemented, and create and enforce new ones to ensure reasonable costs to patients and cap the profit margins of the hospitals. The health insurance scheme for central government employees in India purchases services from the private sector. This model can be expanded to scale for the population using the newly launched national health insurance scheme (Ayushman Bharat). 

Adopt Digital Technologies at Scale

In India, as in the US, digital technology is being used to facilitate its vaccination program. A digital track and trace system, named eVin, has been developed to track vaccine availability and facilitate cold chain maintenance for its national immunization program. It is now enjoying widespread use to accelerate Covid-19 vaccination. Online Training Management Information Systems have been indigenously developed. These systems help schedule training for new personnel, maintain records and dashboards facilitate monitoring, evaluation and policy formulation. Medical devices, mobile health apps, and wearable, trackable technology have all enhanced opportunities for personalized care, self-management, behavior modification and tele-monitoring of patients [43,44].

India should expand such application of technology to other portfolios within the health sector, as has been done by the High-Income Countries, where innovations in digital technology are supporting the delivery of vital healthcare. The COVID-19 pandemic has catalyzed the pace and scale of this process and enabled the delivery of healthcare to populations who would have been denied such care otherwise. The uptake of telemedicine for consultations with healthcare providers increased 50 to 175 times in the US during the COVID-19 pandemic [45]. The scope of medical examination and procedures has expanded markedly during this pandemic. AI-embedded logarithms now diagnose COVID-19 disease from chest x-rays and CT scans. Doctors can now perform an ophthalmic fundal examination online. Standards for conducting remote orthopedic examinations are now used successfully. Drones are being used to deliver medicines to communities [46]. A shortage of protective equipment during the pandemic was overcome by manufacturing it locally using 3D printing [47].

India must close its digital divide if it is to take advantage of digital technology to aid the delivery of healthcare to the rural areas where it is needed the most. India has an internet density of 25.3% for its rural areas where 66% of its population resides and an internet density of 97.9% where 34% of its population lives [48]. The private health sector is taking a lead in this area, with some centers enlisting a cognitive platform for the delivery of healthcare in oncology [49], but the reach of such initiatives is very limited. Market-driven forces cannot be trusted to meet the needs of an entire nation. Government must step in and direct the effort. Telemedicine can amplify the reach of care, thereby helping decrease maternal and child mortality, which are among the highest in the world in India. National policy must make this a priority. The use of digital technologies at scale can aid in the control of risk factors for non-communicable diseases such as smoking, alcohol abuse, physical inactivity, and unhealthy diet [50]. There is an urgent need for this, for India is already known as the diabetes capital of the world [51]. Increasing digital literacy can aid delivery and monitoring of care for the elderly. It can ensure privacy in accessing care for those with mental health issues, thereby helping overcome the stigma associated with mental health in the country [52]. But this will only happen if the central government again asserts leadership in the fight for better health for all Indians, not just those with the money to pay unassisted.

India needs to have a live register for health personnel and infrastructure. For instance, India does not have data about the exact number or geospatial details about its practicing doctors. Having accurate data about the quantity and geospatial location of its manpower will increase the efficiency and effectiveness of its utilization.

Those trained to provide them must be well-equipped to provide the services they have been trained for. Lack of transparency in creating training schedules, coupled with the erratic posting of personnel, all too often results in the same personnel staying at district or state headquarters, where living conditions are generally better than in the underserved regions. Online training can help overcome this challenge. It must be adopted. This will require investment in the necessary infrastructure to do so. A two-year intermittent training initiative for upgrading the skills of nurses was taken by faculty from the University of Nottingham in India. The training received positive evaluations from participants, but few implemented what was learned due to deeper structural problems including national and state-level policies, working conditions, and staff shortages [52]. Sourcing health personnel who are native residents of the communities, providing ongoing training in clinical and social skills, using a non-hierarchical work environment, and providing individualized mentoring help personnel retention and ensure personnel motivation [53].

Increasing violence against healthcare personnel, particularly doctors is a worrisome new trend that must be met head-on. It only compounds a bad situation. A low physician-to-patient ratio not only results in delays in attending patients but adds to costs. Underpaid physicians are tempted to take on more patients than they can reasonably serve. Defensive medicine practices set in. Overwhelmed physicians protect themselves by ordering unnecessary tests and procedures. The profit motive of the private sector forces mandatory advance payments [54]. Unethical practices by pharmaceutical companies [54] and an intense focus on specialization [55] abet these issues, tempting providers to refer unnecessarily and prescribe treatments [55] that may not be needed to assure patients that their concerns are being addressed. Physicians get entangled in a complex web. Dissatisfied, patients may strike out at a system that is failing them. As physicians, seen as authorities, are in direct contact with their patients, they are most accessible and tend to bear the brunt of agitated and frustrated patients and their families. Until and unless physicians, particularly primary care physicians, are adequately compensated, this trend is likely to continue with the consequences noted earlier. The private marketplace will not be able to solve this. Only a thoughtful approach to government planning can reverse the situation.

## Conclusions

The aims, strategy, structure, and service provision by the public and the private health sector are different, and the recommendations made here are an effort to synergize their services for maximizing efficiencies at scale. Focus on transparency, training and retaining health personnel, strengthening health infrastructure, implementing uniform standards, and a broad overarching policy is essential to ensure India’s health system is able to provide affordable, accessible, quality care for its citizens.
